# Low-dose irradiation promotes Rad51 expression by down-regulating miR-193b-3p in hepatocytes

**DOI:** 10.1038/srep25723

**Published:** 2016-05-26

**Authors:** Eon-Seok Lee, Yeo Jin Won, Byoung-Chul Kim, Daeui Park, Jin-Han Bae, Seong-Joon Park, Sung Jin Noh, Yeong-Rok Kang, Si Ho Choi, Je-Hyun Yoon, Kyu Heo, Kwangmo Yang, Tae Gen Son

**Affiliations:** 1 Research Center, Dongnam Institute of Radiological and Medical Science, 40 Jwadong-gil, Jangan-eup, Gijang-gun, Busan, 46033, Republic of Korea; 2 Department of Radiation Oncology, Korea Institute of Radiological and Medical Sciences, Seoul 139-709, Republic of Korea; 3 In silico Toxicology Research Center, Korea Insititute of Toxciology, Daejeon 305-343, Republic of Korea; 4 Department of Biochemistry and Molecular Biology, Medical University of South Carolina, Charleston, SC 29425, USA

## Abstract

Current evidence indicates that there is a relationship between microRNA (miRNA)-mediated gene silencing and low-dose irradiation (LDIR) responses. Here, alterations of miRNA expression in response to LDIR exposure in male BALB/c mice and three different types of hepatocytes were investigated. The miRNome of the LDIR-exposed mouse spleens (0.01 Gy, 6.5 mGy/h) was analyzed, and the expression of miRNA and mRNA was validated by qRT-PCR. Western blotting, chromatin immunoprecipitation (ChIP), and luciferase assays were also performed to evaluate the interaction between miRNAs and their target genes and to gain insight into the regulation of miRNA expression. The expression of miRNA-193b-3p was down-regulated in the mouse spleen and liver and in various hepatocytes (NCTC, Hepa, and HepG2 cell lines) in response to LDIR. The down-regulation of miR-193b-3p expression was caused by histone deacetylation on the miR-193b-3p promoter in the HepG2 cells irradiated with 0.01 Gy. However, the alteration of histone deacetylation and miR-193b-3p and Rad51 expression in response to LDIR was restored by pretreatment with N-acetyl-cyctein. In conclusion, we provide evidence that miRNA responses to LDIR include the modulation of cellular stress responses and repair mechanisms.

Post-transcriptional gene regulation is primarily modulated by RNA-binding proteins (RBPs) and non-coding RNAs (ncRNAs)^1^,^2^,^3^. ncRNAs play roles in transcription regulation, mRNA decay and translation, RNA and protein localization^4^,^5^, and chromosome maintenance and segregation^6^. As one of the many varieties of ncRNAs, highly conserved 18 to 22 nucleotide-long ncRNAs called microRNAs (miRNAs) generally act as negative regulators of post-transcriptional gene expression^7^,^8^,^9^, and they regulate the expression of more than 50% of the human protein-coding genes by promoting mRNA destabilization and translational repression^2^,^10^.

Recent studies have revealed that miRNA is involved in the cellular response to irradiation^11^,^12^,^13^,^14^. These studies demonstrated the effect of irradiation on miRNA expression profiles both *in vitro*^11^,^12^ and *in vivo*^13^,^14^. Exposure to ionizing radiation significantly alters the miRNA expression patterns in normal and cancer cells^15^. Previous studies also revealed that radiosensitivity is modulated through the alteration of miRNA levels^16^,^17^,^18^. For example, the over-expression of miR-7 prolongs radiation-induced γ-H2AX foci formation and reduces the expression of DNA-dependent protein kinase (DNA-PK) in human cancer cells^16^. In addition, miR-221 and miR-222 directly target phosphatase and tensin homolog (PTEN) and efficiently affect downstream biological processes in tumors. These processes include cell growth, invasion, migration, and radiosensitivity^17^. miR-101 targets the 3′UTRs of DNA-PK and ataxia telangiectasia mutated (ATM) mRNAs to influence both the non-homologous end joining (NHEJ) and homologous recombination (HR) DNA damage repair processes and to sensitize tumor cells to radiation^18^. Thus, by down-regulating key factors in the radiation-related signal transduction pathways, miRNAs play crucial roles in the regulation of radiation responses in tumors. Although transcriptome studies have been conducted on cells exposed to acute radiation^14^,^19^ and mouse models exposed to high doses of radiation^20^, the responses of global miRNA expression profiles to low-dose irradiation (LDIR) have not been thoroughly investigated. In addition, the cellular responses to LDIR, such as their adaptive responses or bystander effects, suggest that LDIR has different characteristics than high-dose radiation. Here, we present the miRNA profiles of mouse spleens irradiated with 0.01 Gy and investigate the mechanisms regulating the expression of miRNAs and their target genes in response to LDIR.

## Results

### Differential expression of miRNAs in mouse spleens exposed to 0.01 Gy irradiation as determined by a microarray

To explore whether the expression of miRNAs is altered in response to LDIR exposure, we performed a microRNAome analysis using a sham sample or irradiated mouse spleens (Genolution Pharmaceuticals, Inc., Seoul, Korea). Our analysis identified 84 miRNAs that were differentially expressed (44 were up-regulated and 40 were down-regulated by at least 1.5 fold) in the 0.01 Gy irradiated and sham mouse spleen samples (analysis of variance, *p* < 0.05) (Supp. Table 2). The differential expression of miRNAs with a *Q* < 0.05 was considered to be significant^21^. As shown in Supp. Table 3, 0.01 Gy irradiation altered the expression of 10 miRNAs in the mouse spleens. For example, miR-877-5p and miR-5114 were up-regulated, whereas miR-3963, miR-378a-3p, miR-193b-3p, miR-125a-5p, miR-378b, miR-365-3p, let-7e-5p, and miR-712-5p were down-regulated in the 0.01 Gy-irradiated mouse spleens compared with the sham spleens. These results demonstrate that the changes in miRNA abundance occur in response to LDIR in the mouse spleen.

### Validation of microarray data in the mouse spleen and confirmation of miRNA expression in liver, kidney, and lung tissue exposed to 0.01 Gy irradiation

**Figure 1:**
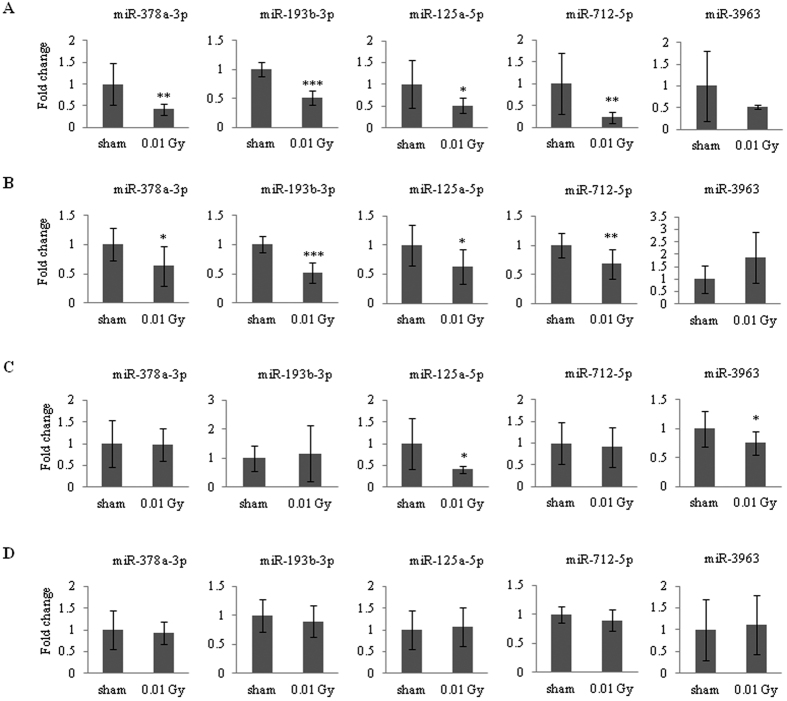
Validation of miRNA expression in mouse tissues in response to irradiation. A qRT-PCR validation of differentially expressed miRNAs (listed in Table 1) was performed. Mice were irradiated with 0.01 Gy (6.5 mGy/h). Six hours post-irradiation, miRNA expression was evaluated in the spleens (**A**), livers (**B**), kidneys (**C**), and lungs (**D**) and reported as fold-change differences relative to compared with the sham-irradiated controls. The mouse U6 and 5S genes were used as the normalization factors. The data shown are the mean ± S.D. (n = 5). Statistically significant differences between the non-irradiated and irradiated samples are indicated (*p < 0.05, **p < 0.01, and ***p < 0.001 vs. the sham group).

The alterations in the expression of the 10 miRNAs described above were validated by qRT-PCR. For the validation, only values below a minimum threshold (Ct < 35) were normalized to avoid artifacts. In our qRT-PCR analysis (Fig. 1A), only 5 miRNAs (miR-378a-3p, miR-193b-3p, miR-125a-5p, miR-712-5p, and miR-3963) generated acceptable Ct values, whereas the other 5 miRNAs (miR-877-5p, miR-5114, miR-378b, miR-365-3p, and let-7e-5p) fell below the minimum threshold because of their lower abundance. Consistent with the results of the microarray analysis, the expression levels of miR-378a-3p, miR-193b-3p, miR-125a-5p, and miR-712-5p decreased in the 0.01 Gy-irradiated mouse spleens compared with the sham group, whereas the expression of miR-3963 did not change significantly. We also confirmed the expression of the 5 miRNAs in liver (Fig. 1B), kidney (Fig. 1C), and lung tissue (Fig. 1D). The alteration of miRNA expression in the liver was consistent with that in the spleen. The levels of miR-378a-3p, miR-193b-3p, miR-125a-5p, and miR-712-5p decreased in response to LDIR, although the level of miR-3963 did not (Fig. 1B). Down-regulation of miR-125a-5p and miR-3963 was observed only in the kidney (Fig. 1C). Interestingly, the abundance of all of the tested miRNAs in the mouse spleen did not change in the lung (Fig. 1D). These results demonstrate that LDIR affects the expression of a subset of miRNAs in a tissue-specific manner.

### Identification of the mechanism that regulates miR-193b-3p expression in HepG2 cells exposed to 0.01 Gy irradiation

**Figure 2:**
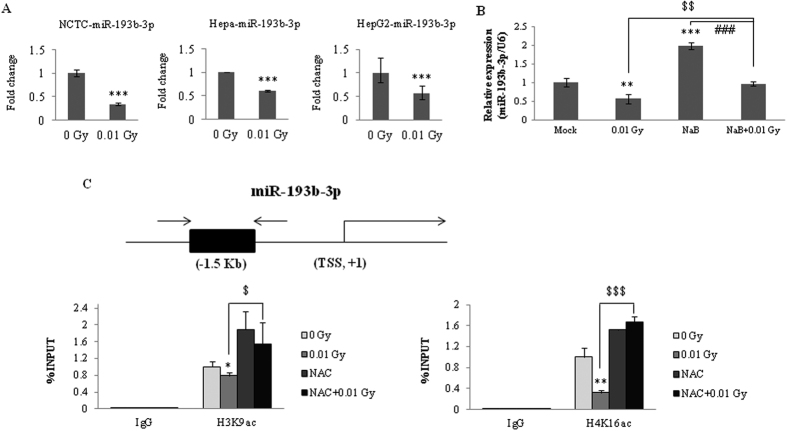
Involvement of histone deacetylation at the miR-193b-3p promoter regions in HepG2 cells exposed to 0.01 Gy irradiation. (**A**) Alterations of miR-193b-3p expression were examined in normal mouse cells (NCTC), mouse hepatoma cells, and human hepatoma cells (HepG2) by qRT-PCR. The cells were irradiated with 0.01 Gy (6.5 mGy/h). Six hours post-irradiation, miRNA expression was evaluated and reported as fold-change differences relative to the sham-irradiated controls. (**B**) When the cells were pretreated with NaB (a histone deacetylase inhibitor) for 24 h, alterations in miR-193b-3p expression were observed in response to irradiation in HepG2 cells. The data were normalized using a mammalian U6 gene and are expressed as the mean ± S.D. (**C**) Chromatin immunoprecipitation (ChIP) assays were performed to evaluate the histone deacetylation of the miR-193b-3p promoter regions in HepG2 cells with or without NAC pretreatment (20 mM) 3 h after exposure to 0.01 Gy irradiation. Chromatin was immunoprecipitated with the anti-H3K9ac, anti-H4K16ac, or anti-IgG antibodies, and DNA was analyzed by qRT-PCR using miR-193b-3p-specific primers. The results are shown as a percentage of the chromatin input. The ChIP samples were quantified by qRT-PCR. The input was used as an internal control, and IgG was used as the antibody control. The statistically significant differences between the non-irradiated and irradiated samples are indicated (*p < 0.05, **p < 0.01, and ***p < 0.001 vs. the non-irradiated control; ^###^*p* < 0.001vs. the NaB-treated group; and ^$^*p* < 0.05, ^$$^*p* < 0.01, and ^$$$^*p* < 0.001 vs. 0.01 Gy irradiation).

The down-regulation of miR-193b-3p after exposure to 0.01 Gy irradiation (Fig. 2A) was confirmed in various liver cell lines, including a mouse normal liver cell line (NCTC), a mouse hepatoma cell line (Hepa), and a human hepatoma cell line (HepG2). Recent studies have shown that a subset of miRNAs is transcriptionally regulated^22^. To determine whether the down-regulation of miR-193b-3p occurs at the transcriptional level, we measured the abundance of miR-193b-3p in cells pretreated with a histone deacetylase inhibitor (HDACi). As shown in Fig. 2B, miR-193b-3p expression was restored to basal levels in the HepG2 cells pretreated with the HDACi sodium butyrate (NaB). In addition, the up-regulation of miR-193b-3p expression observed upon exposure to NaB alone was reduced when the HepG2 cells were treated with a combination of NaB and 0.01 Gy irradiation. To further confirm these results, we next investigated the down-regulation of miR-193b-3p in response to LDIR using ChIP to explore the possible effects of histone deacetylation on the miR-193b-3p promoter (Fig. 2C). The levels of both H3K9ac and H4K16ac on the miR-193b-3p promoter decreased in the 0.01 Gy-irradiated HepG2 cells. However, the levels were restored in cells pretreated with the antioxidant N-actyl-cyctein (NAC) (Fig. 2C). These results indicate that the down-regulation of miR-193b-3p is caused by histone deacetylation and the involvement of mild oxidative stress in histone modification in response to LDIR.

### Investigation of miR-193b-3p target genes in HepG2 cells exposed to 0.01 Gy irradiation

**Figure 3:**
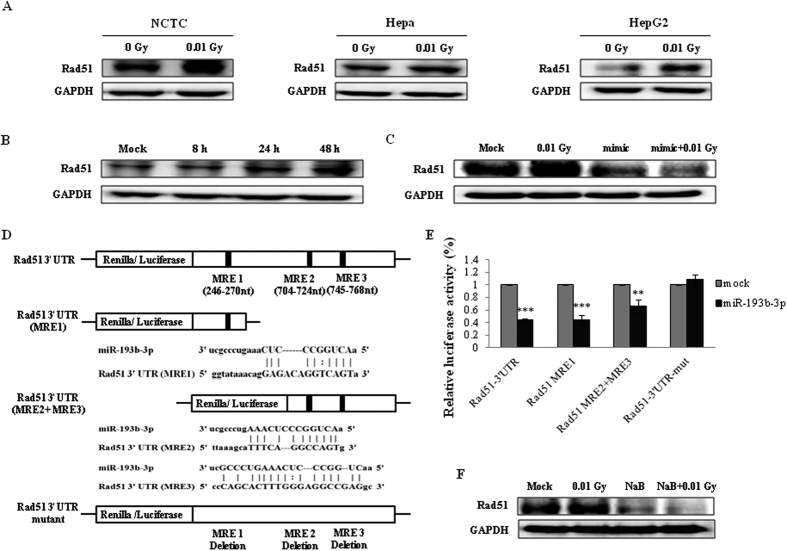
Investigation of miR-193b-3p target genes in the HepG2 cells exposed to 0.01 Gy irradiation. Rad51 protein levels were determined in response to irradiation in the NCTC, Hepa, and HepG2 cells. The cells were irradiated with 0.01 Gy (6.5 mGy/h), and the protein levels were determined 48 h post-irradiation (**A**) and at the indicated time points (**B**). (**C**) Following pretreatment with a miR-193b-3p mimic for 48 h, the Rad51protein levels were observed in response to irradiation in HepG2 cells. (**D**) Diagram of Rad51 mRNA showing the miR-193b-3p binding sites in the 3′untranslated region (UTR). The wild-type or mutant constructs were inserted into the psi-CHECK2 vector directly downstream of the luciferase gene. miR-193b-3p and its predicted seed binding site in the 3′UTR of Rad51 is shown in the top panel; the three miR-193b-3p MREs predicted to be located in the 3′UTR of Rad51 are shown in the middle panel; and the Rad51 3′UTR mutant without a seed binding region for miR-193b-3p is shown in the bottom panel. (**E**) HepG2 cells were co-transfected with either a miR-193b-3p mimic or a negative control (mock) and the Rad51 3′UTR containing wild-type or mutant MREs. Luciferase activity was measured 48 h after transfection using the dual-luciferase reporter assay system. The normalization of renilla luciferase expression was performed using the luciferase gene present on the psiCHECK2 vector. (**F**) Rad51 protein levels were observed in response to irradiation of the HepG2 cells pretreated with the HDACi NaB for 24 h. GAPDH was used as a loading control. Statistically significant differences between the non-irradiated and irradiated samples are indicated (**p < 0.01 and ***p < 0.001).

To identify the mRNAs targeted by miR-193b-3p, we calculated LDIR-specific genome-wide gene expression profiles from GEO microarray datasets and identified 14 potential LDIR-related miR-193b-3p target genes (Supp. Table 4). We focused on genes involved in DNA damage repair, because DNA damage is a main event in response to radiation induced biological effects. Of these 14 genes, *TPX2* and *RAD51* were only associated with DNA damage repair. Moreover, there were no miR-193b-3p biding site on 3′UTR of *ATAD2*, *CDCA2*, *FAM101B* and *TPX2*, when they compared the common target mRNAs from public databases (miRTarBase, miRDB and miRBase). Therefore, we started with Rad51, which plays a pivotal role in homologous recombination for DNA damage repair^22^, for further testing. The Rad51 protein levels were increased in the 0.01 Gy irradiation-treated NCTC, Hepa, and HepG2 cells (Fig. 3A) in a time-dependent manner (Fig. 3B). We also investigated whether Rad51 expression could be regulated by miR-193b-3p by using a miR-193b-3p mimic. As shown in Fig. 3C, the increased levels of Rad51 protein expression were restored in the cells treated with the miR-193b-3p mimic alone and in the cells treated with both the miR-193b-3p mimic and 0.01 Gy irradiation. In addition, we predicted the miR-193b-3p target sites on the Rad51 3′UTR using TargetScan 7.0 (http://www.targetscan.org) and miRTarBase (http://mirtarbase.mbc.nctu.edu.tw). Because the human Rad51 mRNA (NM_0011642) contains 3 miRNA response elements (MREs) targeted by miR-193b-3p (246-270nt MRE1, 704-724 nt MRE2, and 745-768 nt MRE3) (Fig. 3D), we generated luciferase reporter constructs harboring these MREs, the full-length 3′UTR, and mutated MREs after the luciferase coding sequence. Our luciferase assay revealed that treatment with the miR-193b-3p mimic reduced the luciferase activity of the full-length 3′UTR, MRE1, and MRE2+MRE3 constructs compared with that of the mock treatment. However, luciferase activity remained unchanged in the HepG2 cells when the MREs were mutated (Fig. 3E). Finally, we confirmed that the increased Rad51 protein expression observed in the 0.01Gy-irradiated HepG2 cells was abolished by pretreatment with the HDACi (Fig. 3F). Taken together, these results demonstrate that miR-193b-3p directly regulates Rad51 expression in response to LDIR.

### Evaluation of DNA damage and involvement of mild oxidative stress in HepG2 cells exposed to 0.01Gy irradiation

**Figure 4:**
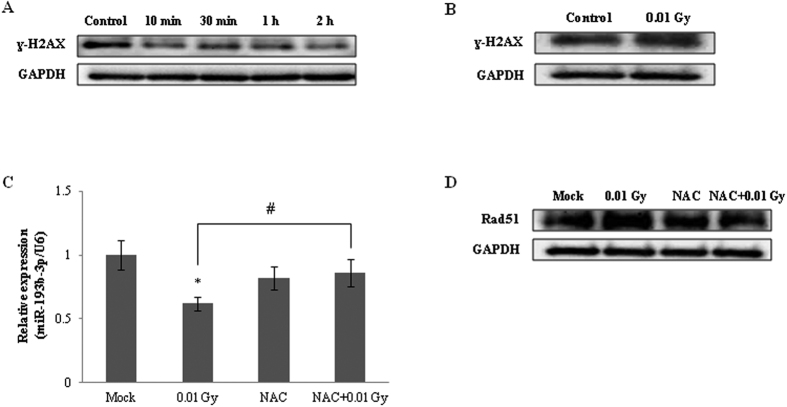
Evaluation of DNA damage and involvement of mild oxidative stress in the HepG2 cells exposed to 0.01 Gy irradiation. γ-H2AX protein levels were detected at the indicated time points (**A**) and 48 h post-irradiation (**B**). The altered expression of miR-193b-3p (**C**) and Rad51 protein levels (**D**) was observed in response to irradiation of the HepG2 cells pretreated with NAC for 24 h. The data were normalized using the mammalian U6 gene and are expressed as the mean ± S.D. GAPDH was used as a loading control. Statistically significant differences between the non-irradiated and irradiated samples are indicated (*p < 0.05 vs. the non-irradiated control and ^#^*p* < 0.05 vs. 0.01 Gy irradiation).

DNA double-strand breaks (DSBs) are the major lethal lesions induced by ionizing radiation; however, DSBs can be efficiently repaired by the DNA repair machinery^23^. The Rad51 recombinase is an essential factor for DNA repair by homologous recombination^22^. To evaluate whether the increased Rad51 expression in the 0.01 Gy-irradiated HepG2 cells was caused by DNA damage, we examined the expression of γ-H2AX at various times post-irradiation. Interestingly, the expression of γ-H2AX decreased 2 h post-irradiation (Fig. 4A), whereas compared with Rad51, it remained unchanged at the 48 h time point (Fig. 4B). These results indicate that the up-regulation of Rad51 expression may be caused by the canonical regulation of Rad51 expression rather than by the activation of DNA repair machinery in response to LDIR. We also tested the involvement of mild oxidative stress in the regulation of miR-193b-3p and Rad51 expression. The results showed that the regulation of miR-193b-3p and Rad51 expression in the 0.01 Gy-irradiated HepG2 cells was restored by pretreatment with NAC (Fig. 4C,D). These results reveal that mild oxidative stress may be involved in the modulation of miRNA expression in response to LDIR.

## Discussion

To accurately transmit LDIR, we developed a novel mouse irradiation apparatus (Supp. Fig. 1). This device allowed for the adjustment of the cage angle and distance from the radiation source, thereby leading to more reliable biological samples for further biological analysis. We performed dose assessments for both the in-house system and the classical system (consisting of a normal-planed shelf on which the mouse cages were placed), using glass dosimeters that were inserted into the heads and abdomens of the mice.

miRNAs modulate the abundance and translation of target mRNAs and play an important role in many biological processes^2^,^10^. Circulating miRNAs (in serum or other body fluids) have been assessed as biomarkers for various diseases, including cancer, cardiovascular and neurological diseases, and several inflammatory and autoimmune diseases^24^,^25^. In addition, current evidence indicates that miRNAs might be involved in the regulation of the LDIR response^26^,^27^. Therefore, we sought to determine whether there are differences in the modulation of miRNAexpression in various tissues from mice exposed to LDIR.

In this study, we identified 5 miRNAs that displayed altered expression in the spleen in response to LDIR and confirmed this altered expression in the liver, kidney, and lung (Fig. 1). To further study the regulation of miRNA expression, we first confirmed the expression of the miRNAs (miR-378a-3p, miR-193b-3p, and miR-125a-5p) in response to LDIR in a normal mouse liver cell line (NCTC), a mouse cancer cell line (Hepa), and a human hepatoma cell line (HepG2). Among the 3 miRNAs, only miR-193b-3p presented an expression pattern in all three cell lines that was consistent with the *in vivo* results (Fig. 2A). The expression of miR-378a-3p was down-regulated in both the NCTC and HepG2 cells, whereas it was up-regulated in the Hepa cells following exposure to 0.01 Gy irradiation (Supp. Fig. 2A). miR-125a-5p was excluded from further studies because its Ct value was greater than 35 in all three cell lines (data not shown).

The biogenesis and function of miRNA have been intensively investigated^2^,^28^,^29^,^30^, and increasing evidence suggests that a subgroup of miRNAs is transcriptionally and epigenetically regulated^31^,^32^. For example, the expression of miR-127 was up-regulated to a greater extent in epigenetically unmasked cancer cells. The DNA methylation level and histone modification (histone acetylation and histone methylation) status at the identified promoter regions of miR-127 presented significant correlations with the expression of mature miR-127^32^. Thus, we also investigated whether the expression of miR-193b-3p is epigenetically regulated in response to LDIR. The expression of miR-193b-3p was up-regulated following pretreatment with the HDACi NaB, and the levels of acetylated histone on the miR-193b-3p promoter were reduced in response to LDIR (Fig. 2). These results indicated that histone deacetylation promotes the alteration of miR-193b-3p expression in response to LDIR. In the case of DNA methylation on the miR-193b-3p promoter, HepG2 cell lines expressed low basal levels of DNMT1^33^. We confirmed that the basal levels of DNMT1 and DNMT3a/ b protein expression and the levels of DNMT1 mRNA in the HepG2 cells were lower than those in the human colonic carcinoma cells (HCT116) (Supp. Fig. 2B,C). In addition, we found that LDIR did not affect the regulation of DNMT expression 3 h and 6 h post-irradiation in the HepG2 cells, whereas it modulated the expression of DNMTs in the HCT116 cells. Based on these results, we did not investigate the possibility of DNA methylation on the miR-193b-3p promoter in response to LDIR in the HepG2 cells. In addition, the possibility of histone methylation on the miR-193b-3p promoter was also excluded from this study. Compared with the decreased miR-193b-3p expression upon exposure to radiation alone, the expression of miR-193b-3p was increased following pretreatment with BIX01294 (a histone methyltransferase inhibitor) alone or in combination with BIX01294 and 0.01 Gy irradiation (Supp. Fig. 2D). Moreover, Rad51 protein expression was not correlated with miR-193b-3p expression or negatively regulated when miR-193b-3p expression increased in response to pretreatment with BIX01294 and 0.01 Gy irradiation (Supp. Fig. 2E). Therefore, the down-regulation of miR-193b-3p expression was not associated with histone methylation on the miR-193b-3p promoter in response to LDIR.

The DNA damage response (DDR) is an essential process that occurs during exposure to ionizing radiation, and DDRs can promote the faithful transmission of the genome in dividing cells by reversing extrinsic and intrinsic DNA damage; therefore, this process is indispensable for cell survival during replication^34^. γ-H2AX acts as the core sensor for the initiation of DDR. Thus, we investigated whether LDIR induced DDR. Interestingly, LDIR decreased γ-H2AX expression within 2 h, whereas the expression of γ-H2AX did not change 48 h post-irradiation (Fig. 4A,B). These results indicate that LDIR activates the DNA damage repair system rather than inducing DNA damage. In this study, we also found that LDIR increased the expression of Rad51, which plays a pivotal role in homologous recombination for DNA damage repair^22^. Other studies have revealed that LDIR induces a beneficial effect on organisms called hormesis^19^,^35^. Despite its potential health risks, LDIR may trigger an adaptive response to exposure to higher levels of irradiation or other toxins by preemptively up-regulating cellular functions, including DNA repair mechanisms^35^.

It has been assumed that the alterations in miRNA expression following LDIR are primarily related to the mild oxidative stress caused by free radicals. The initial mild stress depends on the dose of irradiation, dose rate, and post-irradiation time, and it determines whether the alteration is sustained or terminated, which leads to the control of several gene products involvedin repair, signaling, and communication processes. One study demonstrated that exposure to LDIR (doses between 0.7 and 7.6 cGy) in utero can alter the epigenetic response of agouti viable yellow mice^24^. The authors also determined that the response is partially dependent on the cellular oxidative stress response. Thus, in this study, we examined the regulation of the expression of miR-193b-3p and its target gene, Rad51, and investigated histone deacetylation in the presence of an antioxidant. As expected, the down-regulation of miR-193b-3p expression, up-regulation of Rad51 expression, and decreased deacetylation of histone that were observed in response to LDIR were restored to basal levels in the presence of the antioxidant (Fig. 4C,D).

**Figure 5:**
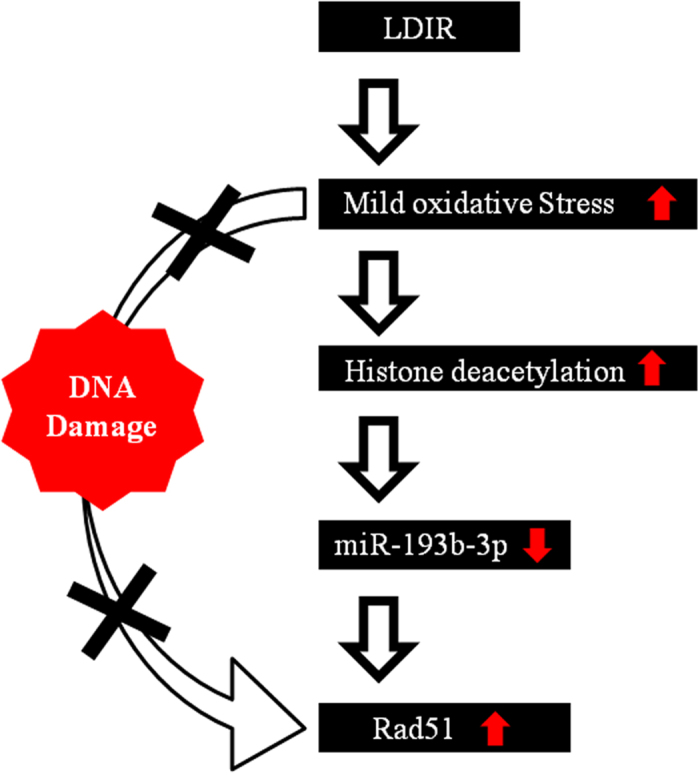
Schematic showing the regulation of Rad51 in response to low-dose radiation. Low-dose radiation induced mild oxidative stress and led to the enhancement of histone deacetylation of miR-193b-3p promoter regions, which caused the down-regulation of miR-193b-3p expression and negatively regulated the expression of the miR-193b-3p target gene, Rad51 (a homolog recombinase), without inducing DNA damage.

In conclusion, miRNA expression profiling was conducted in mouse spleens, livers, kidneys, and lungs following exposure to 0.01 Gy irradiation in this study. We found that the expression of miR-193b-3p was commonly decreased in response to LDIR in the spleen and liver as well as in three liver cell lines. The modification of histone deacetylation on the miR-193b-3p promoter regulated miR-193b-3p expression in response to LDIR. In addition, we showed a correlation between miR-193b-3p and Rad51 expression after treatment with 0.01 Gy irradiation. Our results suggest that miRNAs act together in response to LDIR to mediate pathways required to execute the cellular stress responses and repair mechanisms (described in the schematic in Fig. 5).

## Materials and Methods

### Ethics statement

All the methods were carried out in accordance with the approved guidelines at Dongnam Institute of Radiological & Medical Sciences. All experiment protocols were approved by Dongnam Institute of Radiological & Medical Sciences. Animal studies were conducted in accordance with the guidelines established by the Committee on Use and Care of Animals of Dongnam Institute of Radiological & Medical Sciences. Animals were treated humanely in accordance with the Ministry of Food and Drug Safety on the ethical use of animals.

### Animal and γ-ray irradiation

Male BALB/c strain mice (aged 5 weeks) were obtained from Central Laboratory Animal (Seoul, Korea). Mice were placed in single-housing cages on our newly developed shelf of irradiation apparatus (Supp. Fig. 1; the details are described in the Discussion section). After housing overnight, whole-body irradiations of γ-rays with sham and 0.01-Gy (6.5 mGy/h) exposure were carried out in an irradiation room equipped with a ^137^Cs source (Chiyoda Technol Corp., Tokyo, Japan). The animals were sacrificed 6 h of post-irradiation. Sham mice were also placed in the same cage for overnight without irradiation, and then were sacrificed. Each group comprised 5–6 mice.

### Cells culture

Mouse hepatoma (Hepa-1c1c7) and mouse normal liver (NCTC clone 1469) cell lines were obtained from KCLB (Korean Cell Line Bank, Seoul, Korea). Human hepatoblastoma cell line (HepG2) and human colon carcinoma cell line (HCT116) were obtained from the American Type Culture Collection (ATCC, VA, USA). Hepa-1c1c7 cells were maintained in minimum essential medium (MEM) (WelGENE Inc., Daegu, Korea) supplemented with 10% fetal bovine serum (FBS) (HyClone Inc., Logan, UT) and 1% antibiotic-antimycotic (Gibco, Grand Island, NY, USA). HepG2 cells were maintained in Dulbecco’s modified Eagle medium (DMEM) (WelGENE Inc.) containing 10% FBS (HyClone Inc.) and 1% antibiotic-antimycotic (Gibco). HCT116 cells were maintained in McCoy’s 5A medium (WelGENE Inc.) containing 10% FBS (HyClone Inc.) and 1% antibiotic-antimycotic (Gibco). NCTC clone 1469 cells were maintained in DMEM (WelGENE Inc.) containing 10% horse serum (HyClone Inc.) and 1% antibiotic-antimycotic (Gibco). Cells were maintained at 37 °C in a humidified atmosphere containing 5% of CO_2_.

### miRNA microarray and data analysis

Six hours after whole-body irradiation (0.01 Gy with 6.5 mGy/h) γ-rays or sham exposure, spleens were collected, and the homogenized tissues in isopropanol were sent to an external laboratory for miRNA array analysis (Genolution Pharmaceuticals, Inc., Seoul, Korea).

### miRNA and total RNA isolation and cDNA synthesis

miRNA and total RNA were extracted from using an miRNeasy Mini Kit (Qiagen, Valencia, CA) or Hybrid-R Kit (GeneAll, Seoul, Korea) according to the manufacturer’s protocol. The concentration of miRNA and total RNA were measured using a Nanodrop 2000 spectrophotometer (Thermo Scientific, Wilmington, USA), and 1 μg of miRNA and total RNA were reverse-transcribed using the NCode VILO miRNA cDNA Synthesis Kit (Life Technologies, Carlsbad, USA) or Transcriptor First Strand cDNA Synthesis Kit (Roche, Applied Science, Mannheim, German), respectively.

### qRT-PCR analysis of miRNAs and mRNAs

qRT-PCR was conducted using the CFX96 Touch™ Real-time PCR system (Bio-rad, San Diego, CA), and FastStart Universal SYBR Green Master kit (Roche, Applied Science). PCR was performed using the following conditions: a denaturation program (95 °C for 10 min), an amplification and quantification program repeated 40 times (95 °C for 30 s, 55 °C for 30 s, 72 °C for 40 s with a single fluorescence measurement), a melting curve program (55–95 °C with a heating rate of 0.1 °C per second and a continuous fluorescence measurement) and finally a cooling step to 4 °C. Only values below a minimum threshold (Ct < 35) were normalized to avoid artificial regulation due to sample normalization. The 5S and U6 genes were used for detecting gene amplification and normalization of each sample, respectively. The universal qPCR primer provided as a separate tube in the NCode VILO Kit (Life Technologies) as the reverse primer in the qPCR reaction. Also, the expression level of each mRNA was normalized to that as *GAPDH* and *ACTB* mRNAs. The relative expression of each gene was determined using the comparative threshold (^ΔΔ^Ct) method. All primers are listed in Supp. Table 1.

### Western blot analysis

Total cell lysates containing equal amounts of proteins were loaded onto 4–12% gels for sodium dodecyl sulfate (SDS)-polyacrylamide gel electrophoresis (PAGE) and then transferred to Immobilon-P Transfer Membrane (Millipore, Bedford, MA). The membranes were blocked with 5% non-fat skim milk dissolved in PBS containing 0.02% Tween-20 and incubated overnight at 4 °C with specific primary antibodies. The membranes were subsequently incubated with specific horseradish peroxidase conjugated secondary antibodies. Protein bands were visualized using a Fusion FX5 system (Vilber Lourmat, Eberhardzell, Germany). The following primary antibodies were used: anti-Rad51 (Santa Cruz Biotechnology, CA, USA), anti-DNMT1 (Santa Cruz Biotechnology), anti-DNMT3a (Abcam, Cambridge, MA, USA), anti-DNMT3b (Sigma-Aldrich, St. Louis, MO, USA), anti-gamma H2AX (Abcam), and anti-GAPDH (Advanced ImmunoChemical Inc., Long Beach, CA).

### Chromatin immunoprecipitation (ChIP)

ChIP assays were performed as described previously^36^. The following primers were used: sense ChIP-miR-193b-3p F: 5′-GGGAAAAGAGGCTTTTGGAG-3′ and antisense ChIP-miR-193b-3p R: 5′-CCTCACCCTCCCGAGACT-3′. Anti-H3K9ac (Abcam) and anti-H4K16ac (Abcam) antibodies were used to immunoprecipitate chromatin fragments.

### Luciferase assay

Wild-type or mutant 3′ UTRs of Rad51 were fused to the renilla/luciferase gene using the *Xho*I/*Not*I restriction sites of the psiCHECK2 vector (Promega, Madison, WI) (Fig. 3D). HepG2 cells were co-transfected 20 nM of the miR-193b-3p mimic (Bioneer, Daejeon, Korea) or miRNA mimic negative control (mock) (Bioneer) with 50 ng of psiCHECK2-Rad51 plasmid (wild type-, mutant-, MREs 3′ UTR of Rad51) using lipofectamine 2000 transfection reagent (Invitrogen, Carlsbad, CA). The cells were harvested after 48 h and analyzed for luciferase activity using the dual-luciferase reporter assay system (Promega). The normalization of renilla luciferase expression was performed using the luciferase gene present on the psiCHECK2 vector.

### Reagents analysis

N-acetyl-cystein (NAC) and sodium butyrate (NaB) were obtained from Sigma Chemical Co. (St. Louis, MO, USA). BIX 01294 was obtained from TOCRIS BIOSCIENCE (Ellisville, MO, USA). HepG2 cells (4 × 10^5^ cells) were seeded in 12.5-mm culture T-flasks. The cells were treated with NaB (1 mM), NAC (20 mM) or BIX (1 μM) for 24 h before irradiation. Treated or non-treated control cells were exposed to 0.01 Gy (6.5 mGy/h) γ-rays radiation, and the cells were incubated for 48 h. Target genes protein and mRNA levels were detected by western blotting and qRT-PCR.

### Identification of differentially expressed genes in low dose radiation from GEO dataset

We identified differentially expressed genes (DEGs) in low dose radiation from microarray dataset that are freely accessible in the Gene Expression Omnibus (GEO). We selected dataset of live tissue from mouse (GSE20562). The DEGs were identified by the criteria of a >1.5-fold change, *p*-values < 0.05, and FDR < 5 using the Student’s t-test and FDR analysis.

### Statistical analysis

Quantitative data expressed as the means ± standard deviation. *p*-values less than 0.05 were considered statistically significant using Student’s t-test. The relative levels of miRNA were quantified using the 2^−ΔΔ^CT method, where ^Δ^CT = CT_target_ − CT_reference_.

### False discovery rate (FDR) analysis

Statistical analysis for each gene was performed using Student’s t-test. Additionally, to reduce false positives by t-test, FDR was used with multiple testing corrections. In the analysis, FDR was analyzed as a q-value that measures the proportion of false positives using the Bioconductor q-value package^21^. miRNAs with a q-value less 0.05 (FDR < 5) were selected for experimental evaluation.

## Additional Information

**How to cite this article**: Lee, E.-S. *et al.* Low-dose irradiation promotes Rad51 expression by down-regulating miR-193b-3p in hepatocytes. *Sci. Rep.* **6**, 25723; doi: 10.1038/srep25723 (2016).

## Supplementary Material

Supplementary Information
