# Supplementary material for: Low-dose irradiation promotes Rad51 expression by down-regulating miR-193b-3p in hepatocytes
Source: Sci Rep. 2016 May 26;6:25723. doi: 10.1038/srep25723 (PMC4880923; doi:10.1038/srep25723)
Supplement: Supplementary Information [file srep25723-s1.pdf]

Low-dose irradiation promotes Rad51 expression by down-regulating miR-193b-3p in hepatocytes

Eon-Seok Lee[1,*], Yeo Jin Won[1,*], Byoung-Chul Kim[3], Daeui Park[3], Jin-Han Bae[1], Seong-Joon Park[1], Sung Jin Noh[1], Yeong-Rok Kang[1], Si Ho Choi[1], Je-Hyun Yoon[4], Kyu Heo[1], Kwangmo Yang[1,2,§] and Tae Gen Son[1,§]

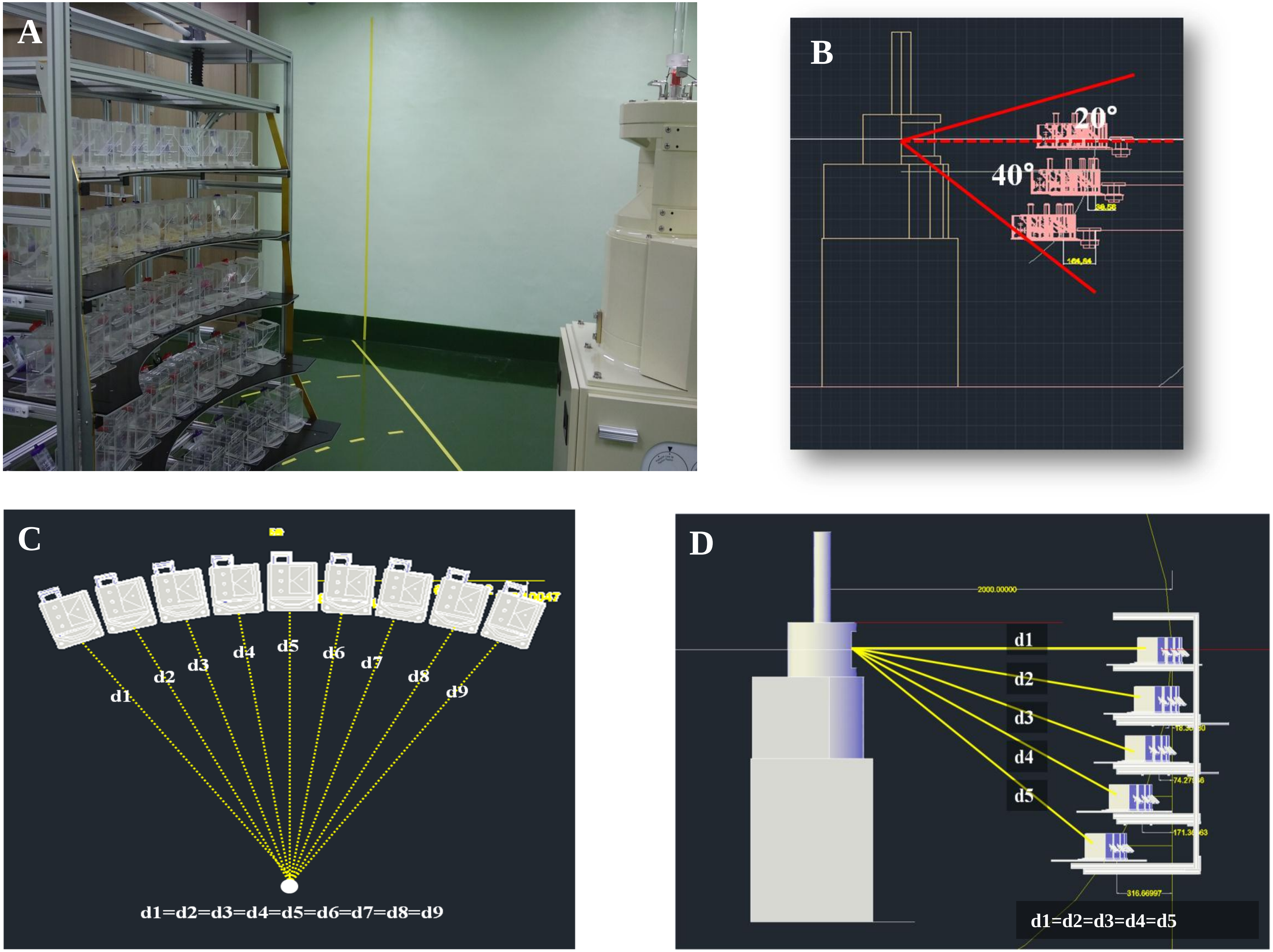

Supplementary Figure.1

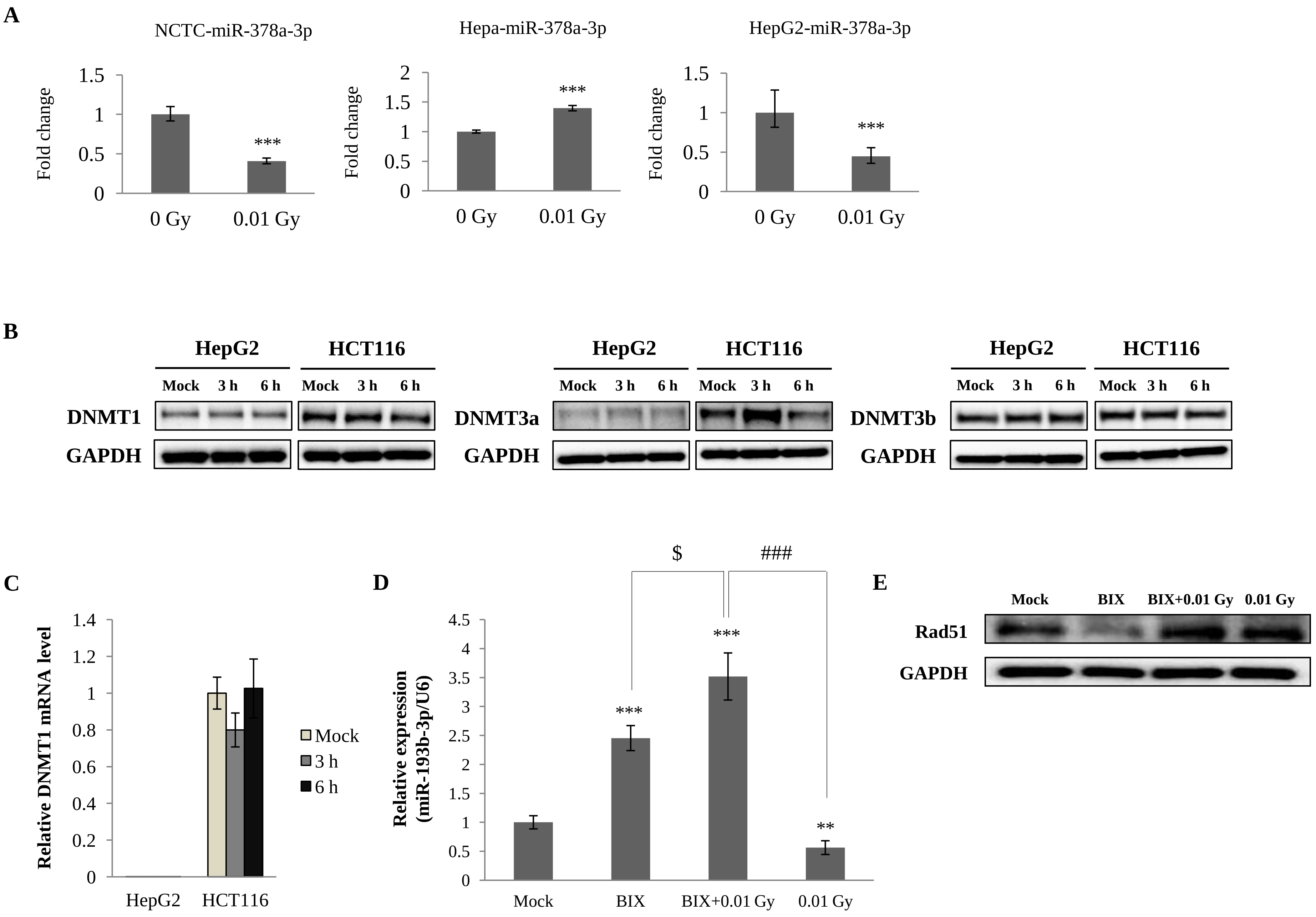

Supplementary Figure.2

Name	Primer sequence	Name	Primer sequence
**Mmu-U6**	**5`-TGGCCCCTGCGCAAGGATG-3`**	**Hsa-U6**	**5`-CGCAAGGATGACACGCAAATTC-3`**
**Mmu-5S**	**5`-GTTAGTACTTGGATGGGAGA-3`**	**Hsa-5S**	**5`-TGGGAATACCGGGTGCTGT-3`**
**Mmu-miR-3963**	**5`-TGTATCCCACTTCTGACAC-3`**	**Hsa-miR-378a-3p**	**5`-ACTGGACTTGGAGTCAGAAGGC-3`**
**Mmu-miR-378a-3p**	**5`-ACTGGACTTGGAGTCAGAAGG-3`**	**Hsa-miR-193b-3p**	**5`-AACTGGCCCTCAAAGTCCCGCT-3`**
**Mmu-miR-193b-3p**	**5`-AACTGGCCCACAAAGTCCCGCT-3`**	**Hsa-miR-125a-5p**	**5`-TCCCTGAGACCCTTTAACCTGTGA-3`**
**Mmu-miR-125a-5p**	**5`-TCCCTGAGACCCTTTAACCTGTGA-3`**	**Hsa-GAPDH F**	**5`-GAAATCCCATCACCATCTTCCAGG-3`**
**Mmu-miR-712-5p**	**5`-CTCCTTCACCCGGGCGGTACC-3`**	**Hsa-GAPDH R**	**5`-GAGCCCCAGCCTTCTCCATG-3`**
		**Hsa-ACTB F**	**5`-AGCGAGCATCCCCCAAAGTT-3`**
		**Hsa-ACTB R**	**5`-GGGCACGAAGGCTCATCATT-3`**
		***Xho Ⅰ*-Rad51-F1**	**5`-GGCTCGAGCCAGGATAAAGCTTCCGGGA-3`**
		***Not Ⅰ*-Rad51-R1**	**5`-GGGCGGCCGCACCCCTCCTCCAAAACCAAA-3`**
		***Xho Ⅰ*-Rad51-F2**	**5`-GGCTCGAGCCCTTCACCATCTACCTGCT-3`**
		***Not Ⅰ*-Rad51-R2**	**5`-GGGCGGCCGCTGGACTTAAAACTCCTGGGCT-3`**
		**ChIP-miR-193b-3p F**	**5`-GGGAAAAGAGGCTTTTGGAG-3`**
		**ChIP-miR-193b-3p R**	**5`-CCTCACCCTCCCGAGACT-3`**
		**DNMT1 F**	**5`- GCACAAACTGACCTGCTTCA-3`**
		**DNMT1 R**	**5`- GCCTTTTCACCTCCATCAAA-3`**

Supplementary Table.1

Up-regulated

NO.	miRNA	F/C	*p*-value
1	mmu-miR-144	5.920	0.022000
2	mmu-miR-877-5p	2.477	0.000807
3	mmu-miR-449a*	2.397	0.013400
4	mmu-miR-3106	2.326	0.006110
5	mmu-miR-1907	2.209	0.016000
6	mmu-miR-5114	2.091	0.000318
7	mmu-miR-1186b	2.046	0.015100
8	mmu-miR-142-3p	2.039	0.044700
9	mmu-miR-1306-3p	1.963	0.004030
10	mmu-miR-3067*	1.960	0.008190
11	mmu-miR-511-3p	1.858	0.000830
12	mmu-miR-208a-5p	1.840	0.019300
13	mmu-miR-375	1.781	0.007110
14	mmu-miR-455*	1.758	0.002520
15	mmu-miR-713	1.751	0.007960
16	mmu-miR-218-1*	1.747	0.009310
17	mmu-miR-487b*	1.731	0.010100
18	mmu-miR-763	1.702	0.016500
19	mmu-miR-700	1.679	0.011300
20	mmu-miR-5119	1.676	0.000199
21	mmu-miR-129-5p	1.630	0.031000
22	mmu-miR-710	1.624	0.033500
23	mmu-miR-3074-2-3p	1.616	0.011000
24	mmu-miR-3093-3p	1.616	0.002840
25	mmu-miR-134*	1.608	0.035000
26	mmu-miR-1931	1.605	0.027100
27	mmu-miR-3059*	1.604	0.003510
28	mmu-miR-1893	1.591	0.029200
29	mmu-miR-3098-5p	1.576	0.002790
30	mmu-miR-362-3p	1.570	0.017400
31	mmu-miR-3091-5p	1.562	0.009400
32	mmu-miR-1198-3p	1.547	0.010400
33	mmu-miR-3075	1.544	0.002970
34	mmu-miR-126-3p	1.542	0.032900
35	mmu-miR-489	1.535	0.034200
36	mmu-miR-322	1.535	0.038900
37	mmu-miR-302c*	1.529	0.018900
38	mmu-miR-3066*	1.525	0.022000
39	mmu-miR-214*	1.520	0.021900
40	mmu-miR-1199*	1.520	0.043300
41	mmu-let-7g*	1.518	0.012800
42	mmu-miR-140	1.517	0.010400
43	mmu-miR-1894-3p	1.511	0.011700
44	mmu-miR-467d*	1.500	0.021000

Down-regulated

NO.	miRNA	F/C	*p*-value
1	mmu-miR-122	12.845	0.05
2	mmu-miR-5100	5.527	0.00556
3	mmu-miR-3963	4.181	0.00131
4	mmu-miR-133a	3.926	0.00913
5	mmu-miR-1a	3.666	0.00556
6	mmu-miR-378a-3p	3.514	0.000163
7	mmu-miR-3472	3.510	0.0356
8	mmu-miR-133b	3.420	0.0234
9	mmu-miR-193b-3p	3.255	0.00103
10	mmu-miR-5115	3.224	0.000249
11	mmu-miR-125b-5p	3.160	0.00405
12	mmu-miR-125a-5p	3.089	0.000575
13	mmu-miR-378b	2.899	0.000953
14	mmu-miR-150	2.279	0.00521
15	mmu-let-7c	2.249	0.00327
16	mmu-miR-3474	2.173	0.00859
17	mmu-miR-365-3p	2.147	0.000788
18	mmu-let-7e-5p	2.137	0.000461
19	mmu-miR-26a	2.093	0.0218
20	mmu-miR-127	2.069	0.00275
21	mmu-miR-712-5p	2.011	2.50E-05
22	mmu-miR-1198-5p	1.950	0.00712
23	mmu-let-7b	1.926	0.00319
24	mmu-miR-222	1.919	0.0149
25	mmu-miR-361	1.879	0.00165
26	mmu-let-7a	1.854	0.0164
27	mmu-miR-212-3p	1.824	0.0115
28	mmu-miR-34a	1.765	0.0416
29	mmu-miR-345-5p	1.756	0.00348
30	mmu-miR-23b	1.730	0.00238
31	mmu-miR-100	1.723	0.00111
32	mmu-miR-326	1.710	0.0172
33	mmu-miR-151-5p	1.704	0.0128
34	mmu-miR-10a	1.703	0.0206
35	mmu-miR-329	1.590	0.0201
36	mmu-let-7d*	1.566	0.00521
37	mmu-miR-331-3p	1.560	0.0252
38	mmu-miR-706	1.528	0.0174
39	mmu-miR-484	1.519	0.00566
40	mmu-miR-29c*	1.501	0.0211

Supplementary Table.2

	miRNA profiles					
	Up-regulated			Down-regulated		
Radiation dose	Name	F/C	*q*-value	Name	F/C	*q*-value
0.01 Gy	miR-877-5p	2.477	0.029035	miR-3963	4.181	0.035044
	miR-5114	2.091	0.018079	miR-378a-3p	3.514	0.015083
				miR-193b-3p	3.255	0.031227
				miR-125a-5p	3.089	0.026149
				miR-378b	2.899	0.030957
				miR-365-3p	2.147	0.029035
				let-7e-5p	2.134	0.023294
				miR-712-5p	2.011	0.011346

Supplementary Table.3

miRNA	PUBMED ID	#ID_REF	GENE	FOLD_CHANGE
Hsa-miR-193b-3p	23622248	GSE20562_mouse_liver_low:1440950	*AKT1*	2.296
Hsa-miR-193b-3p	23622248	GSE20562_mouse_liver_low:1443146	*ATAD2*	3.039
Hsa-miR-193b-3p	20304954	GSE20562_mouse_liver_low:1447363	*BUB1B*	1.91
Hsa-miR-193b-3p	20304954	GSE20562_mouse_liver_low:1459254	*CDCA2*	5.858
Hsa-miR-193b-3p	20304954	GSE20562_mouse_liver_low:1421860	*CLSTN1*	2.495
Hsa-miR-193b-3p	20304954	GSE20562_mouse_liver_low:1456603	*FAM101B*	5.037
Hsa-miR-193b-3p	21512034	GSE20562_mouse_liver_low:1430010	*NCAPD2*	2.902
Hsa-miR-193b-3p	20304954	GSE20562_mouse_liver_low:1423699	*NCAPH2*	3.216
Hsa-miR-193b-3p	20304954	GSE20562_mouse_liver_low:1451698	*PSRC1*	2.448
Hsa-miR-193b-3p	20304954	GSE20562_mouse_liver_low:1418281	*RAD51*	3.406
Hsa-miR-193b-3p	20304954	GSE20562_mouse_liver_low:1439855	*TMTC1*	6.217
Hsa-miR-193b-3p	23622248	GSE20562_mouse_liver_low:1447518	*TPX2*	1.531
Hsa-miR-193b-3p	21512034	GSE20562_mouse_liver_low:1415978	*TUBB3*	5.201
Hsa-miR-193b-3p	23622248	GSE20562_mouse_liver_low:1454281	*XPO7*	5.204

Supplementary Table.4

Supplementary Figures Legends

Supplementary Figure 1. Photograph of the ^{137}Cs gamma-ray irradiation system.
(A) Overview of the irradiation apparatus, which consists of an irradiator and an animal shelf. (B) CAD drawing of the beam collimator angle (the top and bottom irradiation angles are 20°and 40°, respectively). Adjustments to the distance from the irradiation source at the same level (C) and at different levels (D) are indicated.

Supplementary Figure 2. Involvement of DNA and histone methylation in the response to 0.01 Gy irradiation in HepG2 cells.
(A) Alterations to miR-378a-3p expression were examined in normal mouse cells (NCTC), mouse hepatoma cells, and human hepatoma cells (HepG2) by qRT-PCR. The cells were irradiated with 0.01 Gy (6.5 mGy/h) irradiation. Six hours post-irradiation, the miRNA expression levels were evaluated and reported as fold-change differences relative to the sham-irradiated controls. DNMT1, DNMT3a, and DNMT3b protein levels (B) and DNMT1 mRNA levels (C) were measured 3 h or 6 h post-0.01 Gy irradiation in the HepG2 and HCT116 cells by western blotting and qRT-PCR, respectively. Following a 24 h pretreatment with BIX (a histone methylation inhibitor), alterations in the expression of miR-193b-3p (D) and Rad51 protein (E) were observed in response to irradiation of the HepG2 cells. The data were normalized using the mammalian U6 gene and are expressed as the mean±S.D. GAPDH was used as a loading control. Statistically significant differences between the non-irradiated and irradiated samples are indicated ($^{**}p<0.01$ and $^{***}p<0.001$ vs. the non-irradiated control; $^{\$}p<0.05$ vs. the BIX-treated group; and $^{\#\#\#}p<0.001$ vs. BIX plus 0.01 Gy irradiation).

Supplementary Table Legends

SupplementaryTable 1. All of the primers used for the qRT-PCR assays.
Supplementary Table 2. Microarray results detecting 44 up-regulated and 40down-regulated miRNAs in the mouse spleens irradiated by 0.01 Gy. F/C: fold-change.
SupplementaryTable 3. Lists of miRNAs re-analyzed by the false discovery rate (FDR).
The lists of miRNAs (Supp. Table 2) were re-analyzed using the false discovery rate ($Q<0.05$), and new candidate miRNAs were selected for qRT-PCR. Two up-regulated and 8 down-regulated miRNAs were selected. F/C:fold-change.
Supplementary Table 4. Low-dose radiation-specific genome-wide gene expression profile from GEO microarray datasets.
